# On the Mechanism of the Steady-State Gamma Radiolysis-Induced Scissions of the Phenyl-Vinyl Polyester-Based Resins

**DOI:** 10.3389/fchem.2021.803347

**Published:** 2022-01-11

**Authors:** Lorelis González-López, Logan Kearney, Christopher J. Janke, James Wishart, Nihal Kanbargi, Mohamad Al-Sheikhly

**Affiliations:** ^1^ Materials Science and Engineering Department, University of Maryland, College Park, MD, United States; ^2^ Oak Ridge National Laboratory, Chemical Sciences Division, Oak Ridge, TN, United States; ^3^ Brookhaven National Laboratory, Chemistry Department, Upton, NY, United States

**Keywords:** unsaturated polyester resins, gamma radiolysis, EPR spectroscopy, polymer networks, polymer degradation

## Abstract

The major societal problem of polymeric waste necessitates new approaches to break down especially challenging discarded waste streams. Gamma radiation was utilized in conjunction with varying solvent environments in an attempt to discern the efficacy of radiolysis as a tool for the deliberate degradation of model network polyesters. Our EPR results demonstrated that gamma radiolysis of neat resin and in the presence of four widely used solvents induces glycosidic scissions on the backbone of the polyester chains. EPR results clearly show the formation of alkoxy radicals and C-centered radicals as primary intermediate radiolytic products. Despite the protective role of the phenyl groups on the backbone of the radiation-induced polyester chains, the radiolytic-glycosidic scissions predominate. Among the following three solvents used in this study (water, isopropyl alcohol, and dichloromethane), the highest radiolytic yield of glycosidic scission was achieved using water. The •OH radicals produced in the radiolysis of phenyl unsaturated polyester aqueous suspensions very rapidly abstract H atoms from the methylene group, which is followed by a very rapid glycosidic scission. The lowest glycosidic yield was found in the dichloromethane solutions of these polyester resins due to scavenging by the fast electron capture reactions.

## Introduction

Unsaturated polyester resins (UPRs) represent a high-volume class of thermoset materials used in a wide range of coatings, adhesives, and composite applications. (Delahaye et al., 1998; Dholakiya, 2012) These characteristic ester bond-containing reactants can be combined with a myriad of additives to produce a system exhibiting broadly tunable physical and chemical property sets. (Sanchez et al., 2000) Rigidity, thermal stability and chemical resistance are critical design factors and are moderated through the use of various monomers, additives and sub-unit moieties that alter the free volume, glass transitions and chemical interactions. The process for production of polyester resin usually involves the combination of aromatic and unsaturated carboxylic acids or anhydrides with a polyol introduced as an oligomeric, pre-polymer mixture. A reactive diluent, typically a vinyl monomer e.g., styrene, is utilized to reduce the viscosity to allow the components to be easily formed, cast or sprayed. (Huang and Liang, 1996; Lessard et al., 2019) Network formation occurs via the free radical initiated polymerization of the monomer additive, which crosslinks with the unsaturated portions of the chain backbone. (Long, 2014) UPRs can be roughly categorized by the main reactant combinations that largely determine the characteristics of the materials and their optimal application. Terephthalic acid-containing UPRs are the most common for general purpose applications while isophthalic acid or bisphenol-based systems are utilized for higher value applications where superior thermal stability, mechanical properties and chemical resistance are desired. Due to emergent health and environmental associated issues from the widespread use of styrene monomer as a reactive processing aid, new formulations have been in continual development. One promising system that minimizes the use of vinyl monomers employs a combination of maleic anhydride (MA), glycols and dicyclopentadiene (DCPD). (Chiu and Chen, 2001).

Network polyesters comprise an important class of highly cross-linked thermosets that exhibit an essential combination of dimensional stability alongside high chemical and creep resistance. The growing cognizance of the environmental and health impacts of polymer accumulation has created vigorous scientific interest in the delivery of sustainable, circular polymer economies. (Snyder et al., 2018) The fulfillment of these goals will require the development of biodegradable systems that can gradually replace the diverse material property combinations enabled by modern synthetic polymer chemistry. (Gross, 2002; Geyer et al., 2017; Hillmyer, 2017; Fortman et al., 2018) In addition, novel materials and processes must be cultivated to address the abysmal recyclability of commodity polymeric materials. During the 1980s an identification system was introduced that designated six plastic categories to help with sorting to create different pathways for more efficient recycling. (Xanthos, 2012) A seventh category denoted as *other* encompasses specialty polymers and all thermosets. The United States recycles approximately 10% of primary plastic wastes, primarily linear polyethylene terephthalate, and combusts another 16% for energy recovery. (Kerry Thomas, 2007) The molecular structures of thermosets, which endow them uniquely favorable stability, ultimately make reprocess ability impossible. Novel approaches for recyclable thermoset design are showing significant promise, (Gilfrich et al., 1991; Ramis and Salla, 1997; Nowicki et al., 2014; Garcia and Robertson, 2017), however these approaches fail to contend with existing waste streams. While the radiation resistance of network polymers has been studied in the past to ensure operational lifetimes are adequate for selected materials (Nguyen et al., 2018; Hong and Chen, 2019; Sabu Thomas, 2019), radiolysis as an approach to selectively deconstruct macromolecules has not been extensively studied.

In this work, we utilize ionizing gamma (γ) radiation to interact with representative cross-linked polyester networks (Table 1). Our central hypothesis is that the common ester linkages in these thermoset systems can be activated towards radiolysis utilizing catalytic effects arising from the presence of chemically active species from the solvents and thermodynamics of the polymer and solvent interaction. Four representative, commodity grade polyester resins are utilized in the study to understand the effects of constituent composition. The compositions studied here all contain maleic anhydride, polyols and varying amounts of vinyl monomers but are delineated by unique additives DCPD, bisphenol-A (BPA), isophthalic acid and terephthalic acid. The cross-linked resins were subjected to a γ radiation environment at equivalent dosages while submerged in solvent. Spectroscopic results, combined with mechanical testing, show significant contrasts based on the material composition and solution environment. These results outline potential pathways toward deconstruction of thermosets to useful chemicals and products.

**TABLE 1:** Chemical structure and main components of the four polyester-based resins used in this study.

Resin	Main components
Dicyclopentadiene Unsaturated Polyester (DCPD-UPR) 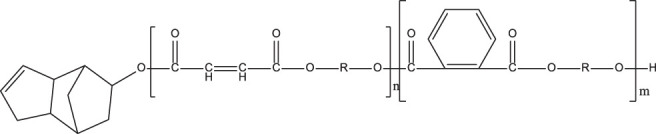	Dicyclopentadiene, Maleic Anhydride, glycols, small amount of vinyl monomers (MMA, vinyl toluene)
Isophthalic Polyester (IP) 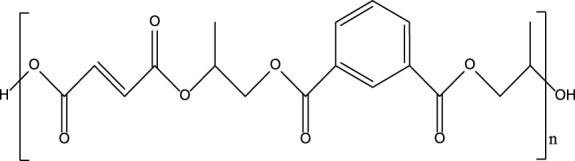	Maleic anhydride, phthalic anhydride, isophthalic acid, propylene glycol
Epoxy Vinyl Ester (EVE) 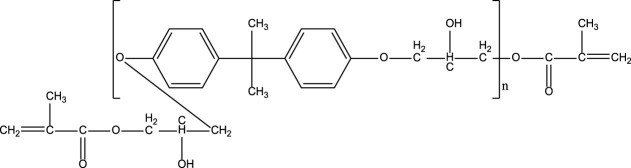	Bisphenol-A, maleic anhydride, small amount of polyols
Terephthalic Polyester (TP) 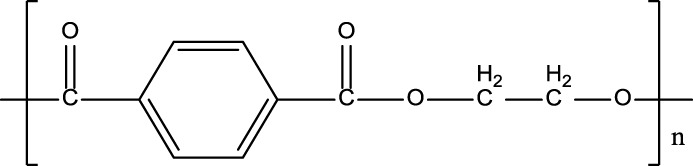	Maleic anhydride, phthalic anhydride, terephthalic acid, propylene glycol

## Materials and Methods

*Materials* Vinyl ester resin samples were provided as approximately 3 mm thick pre-cured plates (AOC resins. Collierville, TN) which were sectioned into bulk specimens and milled into powders for various testing regimens. Dichloromethane (DCM) and isopropyl alcohol (IPA) were purchased from Sigma Aldrich and used as received. Milli-Q (Millipore, Billerica, MA, United States) Type 3 water was used in all experiments in aqueous environments. Solvents were selected on the basis of their compatibility with ionizing radiation.

### Gamma Irradiation

The ^60^Co gamma source in the Chemistry Division of Brookhaven National Laboratory was used for the irradiation of the polyester resins. The samples were irradiated to a total dose of 1,000 kGy, with a dose rate of 6.0 kGy/h.

### Electron Paramagnetic Resonance

All electron paramagnetic resonance (EPR) measurements were performed on a Bruker EMX EPR spectrometer using the following instrument parameters: varying microwave frequency around 9.5 GHz, microwave power of 5.0 mW, frequency modulation of 100 kHz, modulation amplitude of 3.12 G, receiver gain of 6.32 × 10^3^, center field at 3350 G, sweep width of 1000 G, conversion time of 40.96 ms, and time constant of 20.48 ms. Four scans were added to improve signal-to-noise ratio. Instrument software was used to integrate the data and determine radical concentration.

Equation 1 was used to predict the number of EPR lines expected as a result of the hyperfine splitting of radicals.

$$n_{\mathrm{EPR}}=\Pi_{i}\left(2k_{i}I_{i}+1\right)$$ (1)

$$\begin{array}{l} n_{EPR}-total\;number\;of\;EPR\;lines\\ k-equivalent\;nuclei\\ I-spin\;state\\ i-runs\;over\;the\;groups\;of\;equivalent\;nuclei \end{array}$$

### Dynamic Mechanical Analysis

(Jeschke, 2016) Samples of approximately 35 mm long, 5 mm wide and 3 mm thick were sectioned from a cured resin plaque and subjected to irradiation in various environments. The irradiated sections were then dried and placed in a Q800 DMA (TA Instruments. New Castle, DE) in single cantilever mode. An oscillatory strain amplitude of 0.1% at a fixed frequency of 1 Hz was applied to the samples while ramping the temperature environment at 3°/min. Glass transition temperature was recorded as the peak of the tan δ (*G”/G’*).

Bulk samples approximately 3 mm thick were subjected to gamma irradiation in both a dry condition and in the presence of water. Dichloromethane was also used as a test environmental condition but was not able to be tested in the DMA instrument due to complete solvent swelling-induced rupture of the bulk material. DMA is a sensitive technique that is used to probe the dynamic response and the various motional transitions of polymeric materials. Compared to a conventional mechanical test, a sinusoidal stress/strain is applied while varying the frequency and temperature, and the response is measured. This technique exhibits orders of magnitude greater sensitivity to polymer relaxations and secondary chain motions as compared to thermal techniques such as DSC. DMA was employed in this study as a way of probing minute changes in polymer chain architecture as a response to various radiation treatments and solvent environments. Storage modulus and tan δ responses for each polyester thermoset formulation are plotted in Figure 5 and Figure 6, respectively. Tan δ is the tangent of the phase angle (δ) for viscoelastic materials and calculated as the ratio between the loss modulus (*G″*) and storage modulus (*G′*). At experimental temperatures below the glass transition (*T*_*g*_) the material is a rigid, glassy solid consistent with low levels of coordinated motion and exhibits a higher stiffness (Figure 5). As the temperature increases the network’s free volume increases and coordinated motions of the polymer chains over larger length scales become possible. The broadness and shape of these transitions lends valuable insight into the distributions of behaviors in the polymer chains that comprise the bulk system. The region of behavior in thermosets above *T*_*g*_ is referred to as the rubbery plateau. In the rubbery plateau, the storage modulus (*G′*) can be related to the molecular weight between crosslinks (M_c_) by the following simple relationship.

G′≌ρRT/Mc

In this form, a measured polymer density (ρ) and gas constant (R = 8.314 kg^∗^m^2^/s^2^^∗^K^∗^mol) can be used to interrogate the relative changes in the number of crosslinks relative to the reference condition. While this approach has well-established drawbacks, it can be used to qualitatively assess the changes in network structure resulting from irradiation in different environments.

## Results and Discussion

### A. Electron Paramagnetic Resonance Spectra of the Four Resins (Dicyclopentadiene Unsaturated Polyester, Isophthalic Polyester, Epoxy Vinyl Ester, Terephthalic Polyester) Studied

Table 1 shows the chemical compositions and other components of the four resins used in this study. The chemical structures of these resins composed phenyl groups, glycosidic bonds, carbonyl groups, and vynil unsaturations.

#### γ- Radiolysis of Dicyclopentadiene Unsaturated Polyester Resins

**FIGURE 1:**
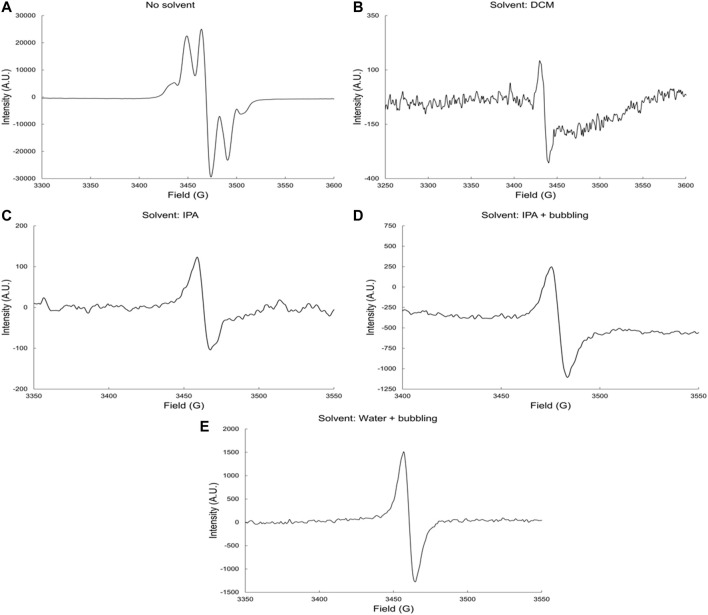
EPR spectra of irradiated DCPD resin **(A)** neat and in **(B)** DCM, **(C)** IPA, **(D)** IPA and bubbling and **(E)** water and bubbling solvents by gamma rays at dose-rate 6.0 kGyh^−1^ to a total dose of 1,000 kGy and in the presence of oxygen.

zThis resin contains radiation polymerizable compounds such as vinyl monomers and toluene. Figure 1 shows the EPR spectra of the irradiated neat DCPD, and its solutions in DMSO, IPA, and H_2_O. The EPR spectrum of irradiated neat DCPD (Figure 1A) exhibits a quartet, which shows clear glycosidic scissions of DCPD. In the presence of all solvents, the EPR (Figures 1B–E) exhibits a singlet rather than the quartet observed in the γ radiolysis of neat DCPD resin. This signal is characteristic of the alkoxyl radicals formed after γ radiolysis of the DCPD resin in DMSO, IPA, and H_2_O.

#### γ-Radiolysis of Isophthalic Polyester Resin

**FIGURE 2:**
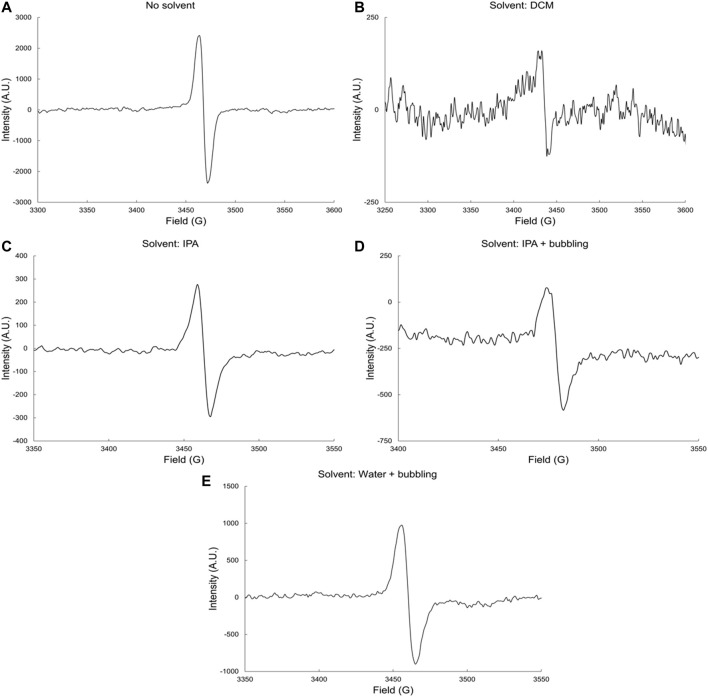
EPR spectra of irradiated IP resin **(A)** neat and in **(B)** DCM, **(C)** IPA, **(D)** IPA and bubbling and **(E)** water and bubbling solvents by gamma rays at dose-rate 6.0 kGyh^−1^ to a total dose of 1,000 kGy and in the presence of oxygen.

Figure 2 shows the EPR spectra of IP resin, irradiated both neat and in the presence of DCM, IPA, and H_2_O. The hyperfine splitting observed shows a singlet for all cases. As in the case of DCPD, scission of the glycosidic bonds leads to the formation of alkoxyl radicals, which can be observed by EPR measurements.

#### γ-Radiolysis of Bisphenol-A, Epoxy Vinyl Ester Resin

**FIGURE 3:**
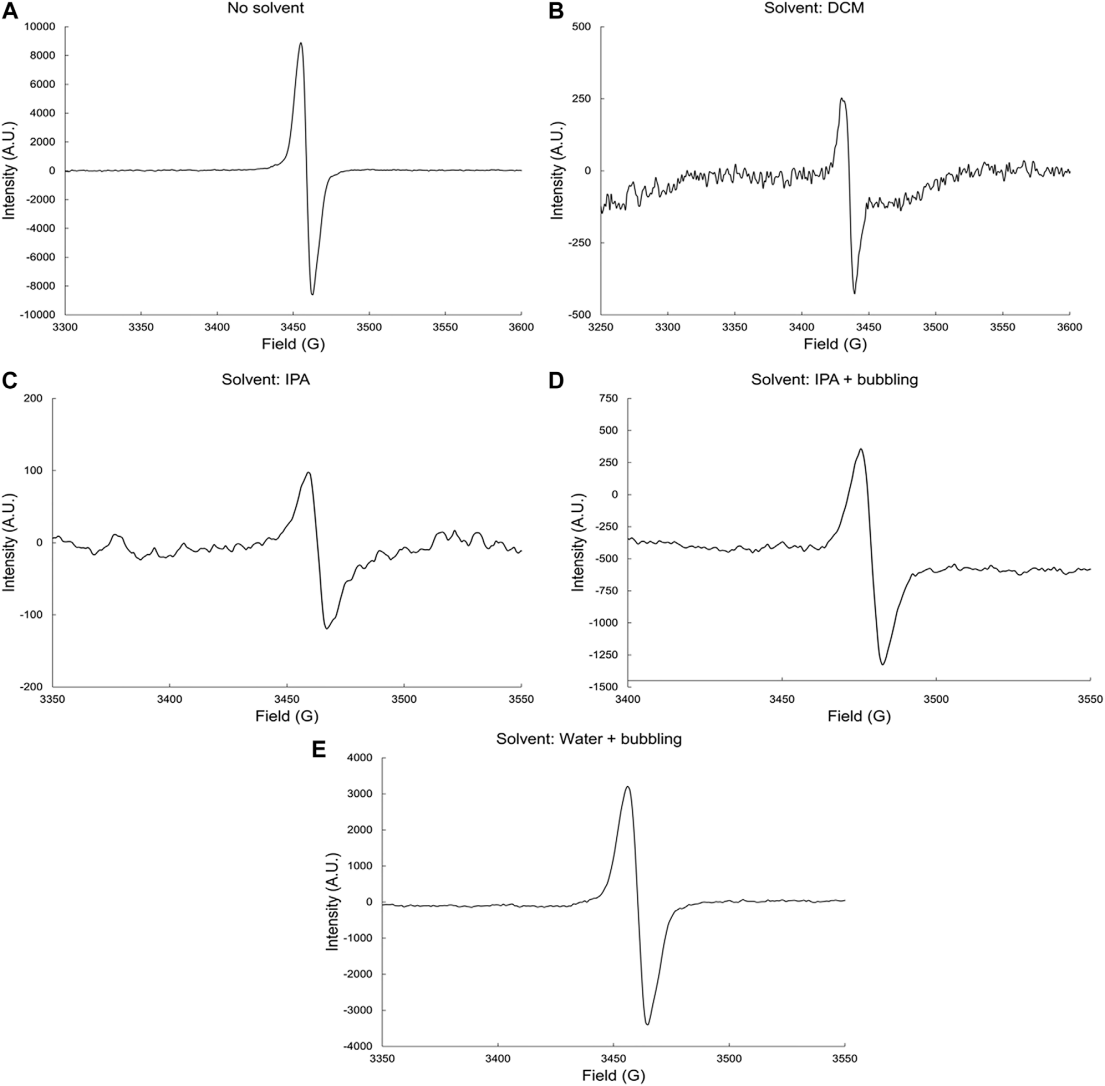
EPR spectra of irradiated EVE resin **(A)** neat and in **(B)** DCM, **(C)** IPA, **(D)** IPA and bubbling and **(E)** water and bubbling solvents by gamma rays at dose-rate 6.0 kGyh^−1^ to a total dose of 1,000 kGy and in the presence of oxygen.

The EPR spectra of EVE resin neat and in the presence of DCM, IPA, and H_2_O solvents, is shown in Figure 3. Similar to the IP resin, γ radiolysis of EVE resin leads to the formation of alkoxyl radicals. This is observed in the EPR spectra, where the signal measured is a singlet for EVE irradiated both neat and in the presence of the various solvents.

#### γ-Radiolysis of Terephthalic Polyester Resin

**FIGURE 4:**
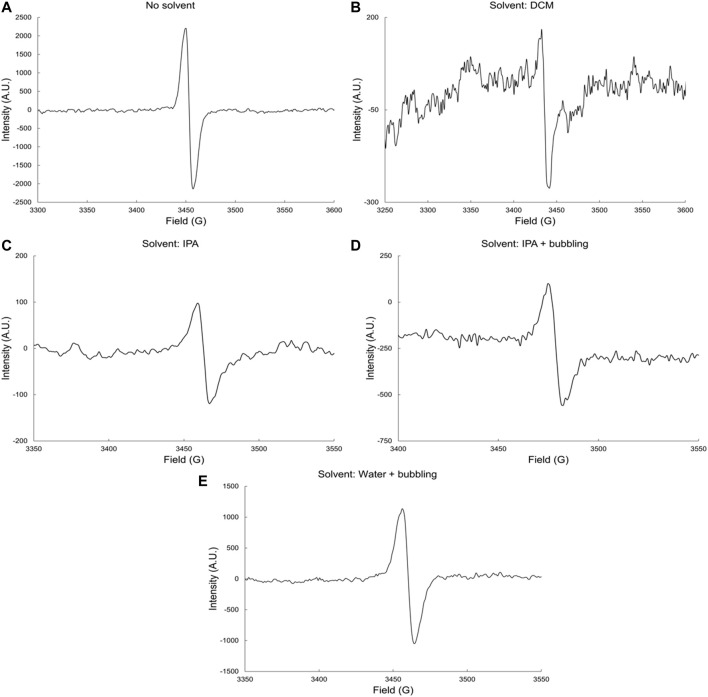
EPR spectra of irradiated TP resin **(A)** neat and in **(B)** DCM, **(C)** IPA, **(D)** IPA and bubbling and **(E)** water and bubbling solvents by gamma rays at dose-rate 6.0 kGyh^−1^ to a total dose of 1,000 kGy and in the presence of oxygen.

Finally, the results for the irradiation of TP resin, both neat and in the presence of DCM, IPA, and H_2_O solvents, are shown in Figure 4. The EPR spectra collected exhibit a singlet in all cases.

### B. Effects of Gamma Irradiation on Polyester Network Polyester Viscoelastic Properties

**FIGURE 5:**
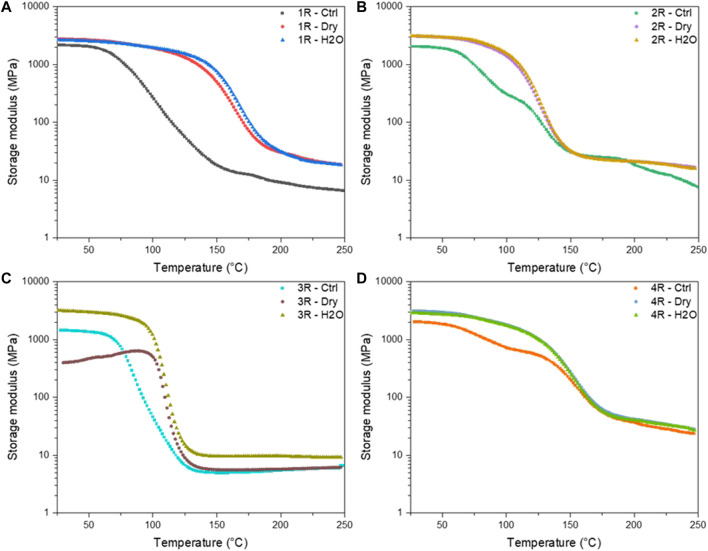
Representative data from dynamic mechanical testing. Resin system **(A)** 1R, **(B)** 2R, **(C)** 3R, **(D)** 4R, showing changes the storage modulus (G′) as a function of temperature and dosing environment. 1R, 2R, 3R, and 4R are DCPD, IP, EVE, and TP resin, respectively.

**FIGURE 6:**
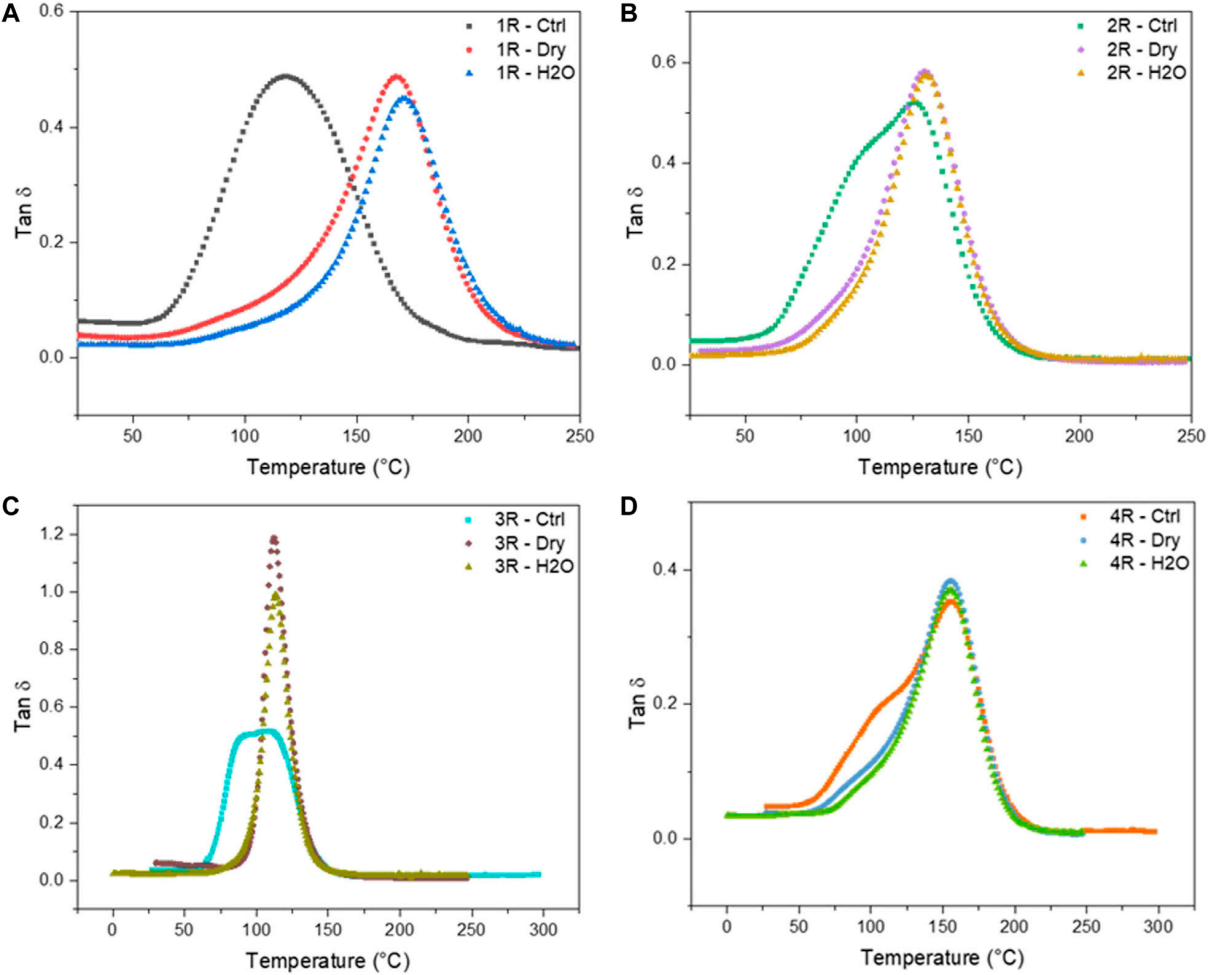
Tan δ as a function of temperature and environment for each resin system, **(A)** 1R, **(B)** 2R, **(C)** 3R, **(D)** 4R, where 1R, 2R, 3R, and 4R are DCPD, IP, EVE, and TP resin, respectively.

Figure 5 summarizes the temperature-dependent storage modulus for each resin composition. Resin DCPD (Figures 5A, Figure 6A) is a low-styrene-content, dicyclopentadiene-based resin formulation. The glass transition temperature (*T*_*g*_) and the rubbery plateau of the DCPD samples shift to significantly higher temperature after irradiating the resin in dry and aqueous environments, both showing nearly identical behaviors. In addition to a large shift upward of the *T*_*g*_, the peak width is also reduced, which is indicative of a reduction in the dispersity of molecular segments. (Menard and Menard, 2015). This is consistent with the resin having a low initial crosslink density that increases after the resin is cured through irradiation, due the presence of residual unsaturated moieties, which are highly susceptible to radiation-induced curing mechanisms. (Abdel-Rahman et al., 2018). The IP, EVE, and TP resins do not exhibit a similar sensitivity to irradiation relative to the DCPD system. As shown in Figure 6B, in the IP resin system we observe a shoulder in the control sample that is eliminated following irradiation, suggesting a loss of the fast-relaxing segments population due to integration into the primary network structure from increased crosslinking. The EVE and the TP resin formulations (Figures 6C,D, respectively) exhibit a similar transition from broader tan δ with apparent shoulders, to a narrower tan δ with a slightly higher *T*_*g*_ and normal distributions. Despite the EPR results which show clear signs of radiolysis in the powder systems, the *T*_*g*_ values of all polyester formulations used in this study (DCPD, IP, EVE, TP) increase with exposure to gamma irradiation in both dry and aqueous environments. This can be explained by the difference in the state of the resin studied by EPR and DMA, where irradiated resin powder was analyzed by EPR and irradiated bulk resin was analyzed by DMA. The components of all these resins include dicyclopentadiene, maleic anhydride, glycols, small amount of vinyl monomers (MMA, vinyl toluene), phthalic anhydride, isophthalic acid, propylene glycol, and terephthalic acid. All these compounds can undergo ionizing radiation-induced polymerization reactions, which seem to dominate the bulk-scale properties relative to the microenvironments probed in EPR. Additionally, the formation of radicals after irradiation discerned using EPR arising from the solvent-mediated effects are expected to be highly diffusively limited and therefore show weak dependence for irradiated materials in the bulk. Characterizing the impacts of gamma irradiation and solvent-activated effects in both a micronized powder and in the bulk via DMA clearly shows a significant dependence of polyester molecular structure and additives on their susceptibility to radiolysis environments.

### C. Proposed Mechanisms for Radiolysis of Neat Resins and in the Presence of Solvents

#### Proposed Mechanism for Radiolysis of Neat Polyester Resin Composites (Dicyclopentadiene Unsaturated Polyester, Isophthalic Polyester, Epoxy Vinyl Ester, Terephthalic Polyester)

**FIGURE 7:**
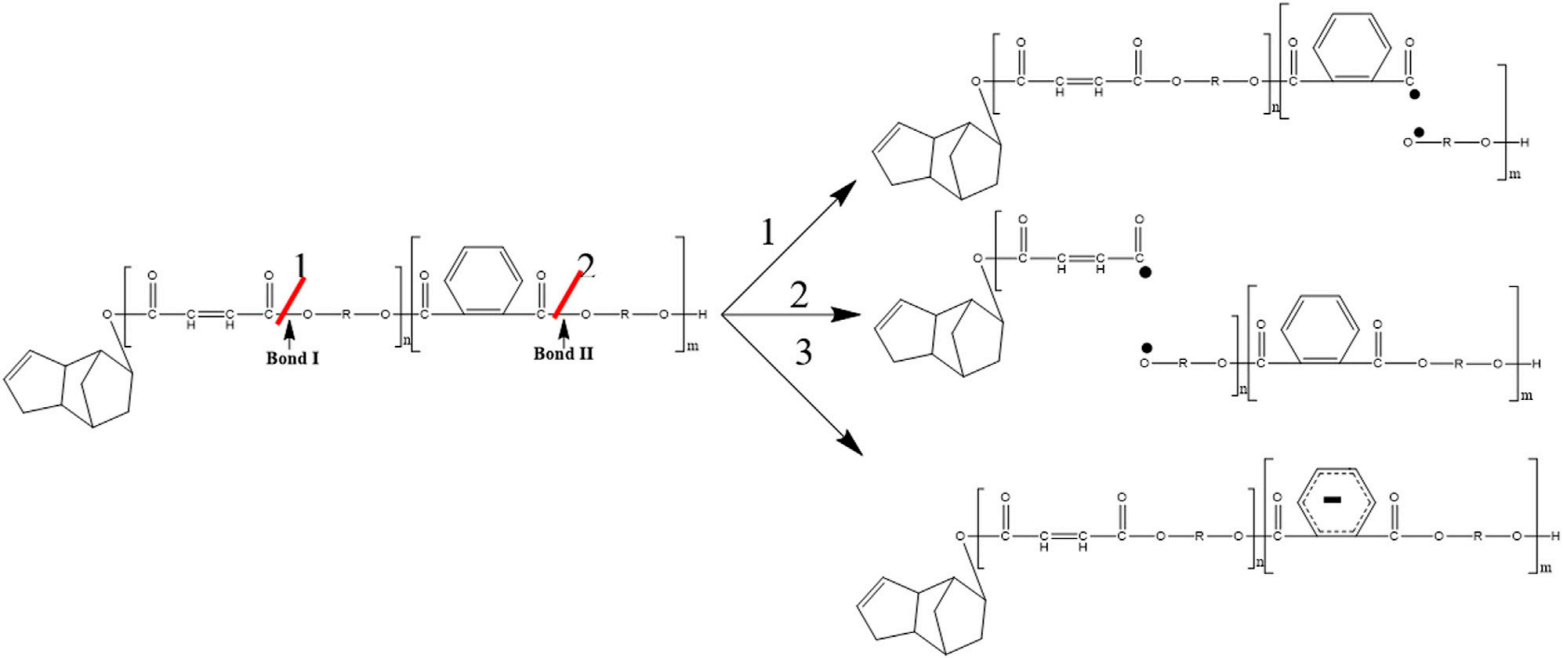
Proposed mechanism for the γ-radiolysis of DCPD resin.

Figure 7 shows the direct γ radiolysis of neat DCPD as a typical unsaturated phenyl ester. As demonstrated by EPR results, the main effect of γ radiolysis is the creation of glycosidic scissions. Figure 1A shows the quartet expected from the hyperfine splitting after the glycosidic scissions. This was confirmed by applying Equation 1 to the radicals generated and shown in the proposed mechanism (Figure 7) as pathways 1 and 2. The calculation of the number of peaks expected from the hyperfine splitting is shown in Table 2. It is expected that the bonds between O-C (bond I) in the *n* component and O-C (bond II) in component *m* are weakened due to the presence of the carbonyl group and oxygen. Based on Equation 1, a quartet is expected from the scission of bond I and a singlet from the scission of bond II. Despite the fact that the γ radiolysis was conducted in the presence of oxygen, it is not expected for the oxygen to react with the radicals generated because of their structures. As shown in Figure 7, two alkoxyl radicals are produced and one C-centered radical is produced. Usually, the C-centered radical is expected to react with oxygen. However, in this case the C-centered radical is bonded to the oxygen atom, which prevents the radical from reacting with the O_2_ present in the system. It is expected that the alkoxyl radicals will abstract an H atom from the neighboring molecules, giving rise to the formation of C-centered radicals. This type of C-centered radical reacts with O_2_ to produce the corresponding peroxyl radicals. Pathway 3 shows another expected reaction where the secondary electrons (electrons produced by γ radiolysis via Compton scattering, photoelectric and pair production) add to the phenyl group, producing phenyl radicals. Due to the pi structure of the phenyl radicals, they are relatively stable.

**TABLE 2:** Schematic representation of radical species formed after the radiation-induced scission of polyester chains, and calculation of the number of peaks expected from the hyperfine splitting of each radical.

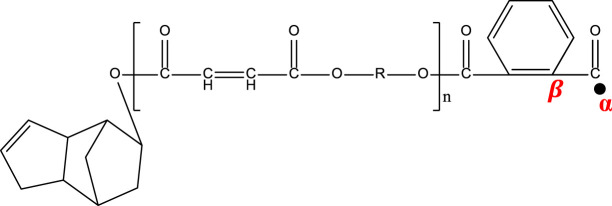	(2(0)+1)(2(0)+1) =1
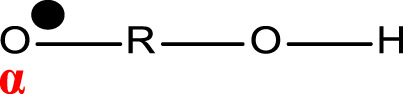	(2(0)+1) =1
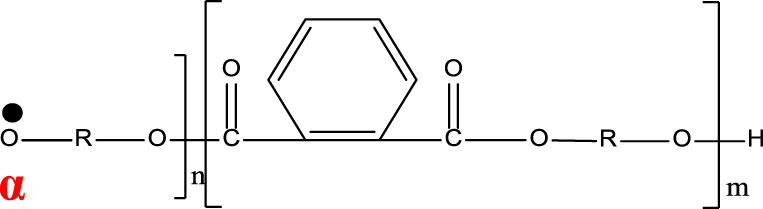	(2(0)+1) =1
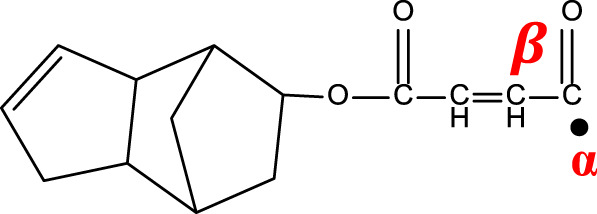	(2(0)+1)(2(1)(1/2)+1) =2
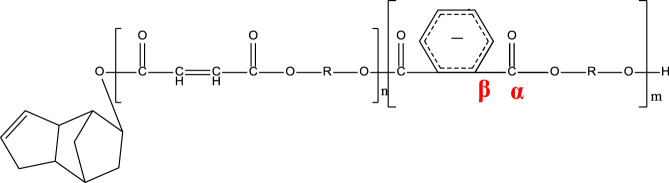	(2(0)+1)(2(0)+1) =1

#### Proposed Mechanism for Radiolysis of Polyester Resin Composites (Dicyclopentadiene Unsaturated Polyester, Isophthalic Polyester, Epoxy Vinyl Ester, Terephthalic Polyester) in the Presence of Solvents

The early events of the radiation chemistry of the DCPD, IP, EVE, and TP in solvents are quite different from the radiolysis without solvents. A large fraction of the radiolytic energy deposition and initial reactive intermediate formation occurs in the solvent rather than the resin. In addition, we used different solvents in this work, which will result in different combinations of radiolytically-produced free radicals and ions that would have a major impact on the radiolytic modification of the polyester resins.

##### γ Radiolysis of Dicyclopentadiene Unsaturated Polyester, Isophthalic Polyester, Epoxy Vinyl Ester, Terephthalic Polyester Aqueous Solutions

H_2_O is the best solvent to start with since it plays a huge role in the radiolysis of this system. Adding a small amount of alcohol can facilitate the diffusion of water through the bulk of the resins. The radiolysis of water by γ rays from ^60^Co produces high yields of the following reactive oxidizing and reducing species:

$$H_{2}O\overset{\gamma }{\to }\;•OH,e_{aq}^{•-},H,H_{2}O_{2},H_{2},H_{3}O^{+}$$

With radiation-chemical yields of: G (^•^OH) = 0.29 µmolJ^−1^, G (e_aq_^•−^) = 0.29 µmolJ^−1^, G (H) = 0.06 µmolJ^−1^, G (H_2_O_2_) = 0.08 µmolJ^−1^, G (H_3_O^+^) = 0.29, and G (H_2_) = 0.04 µmolJ^−1^. (Henglein and Woods, 1991).

Hydroxyl radicals (^•^OH) are highly reactive and it is anticipated that they abstract H atoms from the backbones of the polymer chains and also undergo addition reactions to the double bonds and phenyl groups of the DCPD and other unsaturated phenyl polyesters. These reactions are very fast, with rate constants on the order of 10^8^–10^9^ M^−1^s^−1^. (Tipson and Horton, 1980) In all reactions with the targeted molecules, they produce C-centered free radicals, and initiate the degradation process. To some extent, H-atoms can also exhibit similar behavior, but they are less reactive than ^•^OH radicals. Usually the abstraction of H-atoms from the alpha position to the glycosidic bonds lead to scissions. (von Sonntag et al., 1976) It should be mentioned that H-atoms react with dissolved O_2_ to produce HO_2_^•^, but considering that the pH of the solution is 9.9, HO_2_^•^ is converted very rapidly to O_2_^•−^. So, in summary at this value of pH, no HO_2_^•^ is expected to be present. The reaction between two O_2_^•−^ radicals is negligible. (von Sonntag et al., 1976) O_2_^•−^ is a reductive species and can transfer electrons to the glycosidic sites of the chain.

The aqueous (or hydrated) electron e_aq_^•−^ is a strongly reducing species. It can react with the carbonyl and phenyl groups of the DCPD, and other unsaturated phenyl polyesters through an addition reaction, mainly producing anions. These anions are converted to C-centered radicals through protonation reactions.

e_aq_^•−^ also reacts with dissolved O_2_ very rapidly (rate constant ∼ 1–2 10^10^ M^−1^s^−1^) to produce superoxide radicals (O_2_^•−^). (von Sonntag et al., 1976).

$$e_{aq}^{•-}+O_{2}\to O_{2}^{•-}$$

Although the yield of H_2_O_2_ is relatively low (G (H_2_O_2_) = 0.08 µmolJ^−1^), it contributes to the production of ^•^OH radicals through thermal dissociation.

We conducted our γ radiolysis experiments in the presence of O_2_ to enhance the oxidation-induced scissions of all resins studied in this work. Oxygen reacts very rapidly with DPCD^•^ giving rise to the formation of the corresponding peroxyl radicals (DPCDO_2_^•^). This is a major step towards oxidation and then degradation of the resins. These peroxyl radicals undergo various reactions including self-fragmentation.

##### γ Radiolysis of Dicyclopentadiene Unsaturated Polyester, Isophthalic Polyester, Epoxy Vinyl Ester, Terephthalic Polyester in Dichloromethane Solutions

In the presence of dichloromethane (DCM), all the resins studied showed a decrease in the EPR signal when compared to the γ radiolysis of neat resin and in the presence of other solvents. This reveals a significant decrease in the concentration of free radicals on the resins, which is due to the competition for solvated electrons between the DCM solvent and the polyester resin. Upon irradiation, chlorinated organic compounds undergo dechlorination, proceeding through the following electron capture mechanism: (Henglein and Woods, 1991).

$$CH_{2}Cl_{2}+e^{-}\to •CH_{2}Cl+Cl^{-}$$

The reaction rate constants of the electrons with chlorinated organic compounds range from 10^−9^-10^10^ M^−1^s^−1^. (Neta et al., 1996).

##### γ Radiolysis of Dichloromethane in Isopropyl Alcohol Solutions

At high concentrations, IPA competes very well with the DCPD, IP, EVE, and TP resins for the ionizing radiation. The interactions of ionizing radiation with IPA produces solvated electrons $e_{s}^{-}$ with a G-value of 1-1-1.January 1, 6022 × 10^17^ J, and the following species: (Henglein and Woods, 1991):

$${\left(CH_{3}\right)}_{2}CHOH\;\overset{\gamma \;}{\to }\;\left[{\left(CH_{3}\right)}_{2}CHOH\right]*$$

$$\left[{\left(CH_{3}\right)}_{2}CHOH\right]*\to \left[{\left(CH_{3}\right)}_{2}CHOH\right]++e_{s}^{-}$$

$$\left[{\left(CH_{3}\right)}_{2}\;CHOH\right]+\to {\left(CH_{3}\right)}_{2}\dot{C}HOH+H^{+}$$

$e_{s}^{-}$ are a very reductive species and can react with both the resins and the dissolved oxygen in the mixtures of resin-IPA. $e_{s}^{-}$ attacks the glycosidic bonds on the backbone of the polyester chains, leading to scission and the formation of alkoxyl radicals.

The EPR spectra in Figure 1B, represent the alkoxyl radicals formed directly by γ radiation, and indirectly by the attack of $e_{s}^{-}$ on the glycosidic bonds. In addition, $e_{s}^{-}$ also reacts very rapidly with dissolved O_2_ to produce ${\text{O}}_{2}^{•-}$ . The scavenging of $e_{s}^{-}$ by dissolved O_2_, explains the reduction of the alkoxyl radical concentration, as evidenced by the reduced intensity of the EPR signals. All DCPD, IP, EVE, and TP resin particles were suspended in IPA; no miscible solutions were prepared. Hence, it is expected that $e_{s}^{-}$ attack the surfaces of these suspended resins particles. (Henglein and Woods, 1991).

## Conclusion

Based on the EPR results, it is concluded that gamma rays induce glycosidic scission in the phenyl unsaturated polyester chains in both neat resin and in solvent-resin suspensions. In all cases, oxidizable alkoxyl radicals are produced. Since the alkoxyl radicals are very reactive, they are expected to decay through abstraction of H-atoms from neighboring chains to form C-centered radicals. The relative low irradiation dose rate and the presence of oxygen enhance the decay of the C-centered radicals through peroxidation reactions to produce the corresponding peroxyl radicals. The highest alkoxyl radical yields were achieved in aqueous suspensions of these resins. The relatively high yield of •OH from water radiolysis generates a considerable yield of glycosidic scissions. On the other hand, low alkoxyl radical yields were found in DCM-resin mixtures. DCM very effectively scavenges the secondary electrons, leading to the decrease of the alkoxide yields. All these polyester resins contain free radical polymerizable vinyl monomers such as dicyclopentadiene, maleic anhydride, glycols, small amount of vinyl monomers (MMA, vinyl toluene), phthalic anhydride, isophthalic acid, propylene glycol, and terephthalic acid. The use of 1,000 kGy to induce more scissions on the backbone of the chains, also induces polymerization and even crosslinking of these monomers. In addition, the relatively low irradiation dose rate used in this study also enhances the polymerization reactions of these vinyl monomers. This study shows the combination of mechanical breakdown of the cured bulk in conjunction with gamma radiation may produce a pathway toward the breakdown of the molecular structure of the polyester network.

## Data Availability

The raw data supporting the conclusions of this article will be made available by the authors, without undue reservation.
